# Prospective Assessment of Depression and Anxiety Trajectories Among Emergency Department Patients with Somatic Complaints

**DOI:** 10.5811/westjem.50847

**Published:** 2026-05-14

**Authors:** Mona J. Moukaddem, Mohammed I. Lone, Jorge A. Alarcon, Naman Satsangi, Robert D. Gibbons, Paul I. Musey, David G. Beiser

**Affiliations:** * University of Chicago, Section of Emergency Medicine, Chicago, Illinois; † Indiana University School of Medicine, Department of Emergency Medicine, Indianapolis, Indiana; ‡ University of Chicago, Department of Medicine, Chicago, Illinois

## Abstract

**Introduction:**

Emergency department (ED) patients exhibit higher rates of depression than those in primary care and the general population, but it is unclear whether these symptoms reflect chronic conditions or transient responses to acute stress. Our objective in this study was to evaluate the longitudinal trajectory of depression and anxiety identified in the ED to inform evidence-based screening and intervention strategies.

**Methods:**

Adult, English-speaking ED patients with adequate literacy who presented to two urban academic EDs with somatic (non-psychiatric) chief complaints completed six mental health screening assessments at enrollment. Of 262 approached patients, 188 were enrolled, representing approximately 0.5% of all adult ED visits (188/37,898) during the study period. Follow-up assessments were completed through a secure phone app at one, two, and four weeks after ED discharge. The primary outcome was the longitudinal stability of depression and anxiety symptoms. The secondary outcome was differences in follow-up completion rates by baseline mental health status.

**Results:**

Among 188 patients with baseline assessments, 44 (23%) screened positive for major depressive disorder, 17 (9%) for moderate/severe depression, and 34 (18%) for moderate/severe anxiety at baseline. Overall, 50 patients (27%) screened positive for at least one of these conditions. Follow-up responses at weeks 1 (n = 42, 22%), 2 (n = 41, 22%), and 4 (n = 27, 14%) showed no significant changes in levels of depression as measured by the Computerized Adaptive Test-Depression Inventory or severity of anxiety as per the Computerized Adaptive Test for Anxiety severity. High intraclass correlation coefficients (0.76–0.84) for all measures indicated inter-individual differences accounted for most variance. Stability of the Computerized Adaptive Diagnostic Test for Major Depressive Disorder ranged from moderate to substantial (Cohen kappa: 0.74 at week 1 to 0.46 at week 4). Patients who were positive for major depressive disorder had significantly higher follow-up completion rates at weeks 2 and 4 (*P* = .04).

**Conclusion:**

High baseline rates of depression and anxiety highlight the substantial mental health burden in ED patients. Among those who completed follow-up assessments, severity scores remained stable, suggesting these symptoms reflect ongoing conditions rather than transient stress. Future work should improve follow-up responses and assess whether ED-based identification and treatment improve outcomes.

## INTRODUCTION

Mental health disorders, including major depressive disorder (MDD) and generalized anxiety disorder, are a growing yet under-addressed public health concern, affecting millions worldwide and contributing significantly to global disability. The World Health Organization identifies MDD as the leading cause of disability, accounting for 7.5% of years lived with disability.^1^ These disorders profoundly impact both physical and psychological well-being while imposing a substantial economic burden on healthcare systems through direct and indirect costs.^2^–^4^ Despite their widespread prevalence, depression and anxiety are frequently underdiagnosed and undertreated, especially among vulnerable populations who often seek care in the emergency department (ED).^5^,^6^ This gap in recognition is reflected in administrative data in which psychiatric disorders are absent from the top 20 discharge diagnoses of patients from U.S. EDs, based on *International Classification of Diseases*, 9^th^ Revision, codes.^7^

The ED plays a critical role in the nation’s healthcare system, with approximately 140 million visits annually.^8^ This number has risen as healthcare accessibility declines for many populations, particularly those with unmet mental health needs.^9^ Notably, 23–27% of ED patients with non-psychiatric complaints screen positive for depression and 18–50% for anxiety,^2^,^10^,^11^ rates that significantly exceed the prevalence of these conditions in primary care populations.^12^,^13^ The reasons for this disparity remain unclear but may involve the influence of acute somatic symptoms on screening measures, as depression screening tools tend to yield more false positives in medical populations with concurrent physical symptoms.^14^–^16^ Alternatively, ED-identified depression and anxiety may represent chronic conditions in individuals who rely on the ED due to limited access to healthcare, with these disorders often going unrecognized in primary care, where patients lack consistent follow-up. If this is true, then ED-based diagnosis and intervention could help reduce the burden of untreated mental illness and provide early intervention for those who might otherwise remain undiagnosed and untreated.

A key question is whether positive mental health screening and severity assessments in the ED are affected by measurement bias due to emergency care factors such as acute stress and somatic symptoms, or whether they identify untreated, chronic conditions like major depressive disorder and generalized anxiety disorder. While the short-term test-retest stability of ED-based Computerized Adaptive Testing for Mental Health (CAT-MH) assessments has been demonstrated over a test-retest period of three minutes,^17^ the longitudinal persistence of ED-identified depression and anxiety post-discharge has not been well established. One study found that three-quarters of chest pain patients in the ED with abnormal anxiety symptoms had persistent anxiety at follow-up, suggesting that positive anxiety screenings in the ED are not merely a “white coat” phenomenon.^10^ If positive screenings primarily capture short-term, stress-related responses, routine ED screening alone may not reliably identify individuals in need of longitudinal mental health resources. Conversely, if they indicate persistent conditions, these findings reinforce the case for systematic screening and early intervention in the ED.

**Population Health Research Capsule**

What do we already know about this issue?

*Emergency department (ED) patients have high rates of depression and anxiety, but symptom persistence after discharge is poorly understood*.

What was the research question?

*Do ED-identified depression and anxiety symptoms remain stable over 30 days after discharge?*

What was the major finding of the study?

*Symptoms showed no change over 30 days: intraclass correlation coefficient .76–.84; major depressive disorder agreement κ=0.46–0.74*.

How does this improve population health?

*Findings support ED screening to identify persistent mental health needs and enable referral and early intervention for underserved patients*.

Our objective in this study was to investigate the longitudinal trajectory of depression and anxiety identified in the ED and re-assessed over a 30-day post-discharge period. We hypothesized that ED mental health symptoms reflect chronic mental health disorders rather than temporary responses to acute illness. Specifically, our objectives were to 1) assess the longitudinal test-retest stability of depression and anxiety severity measures, and 2) compare follow-up completion rates among patients who screened positive and negative for these conditions. Ultimately, this study provides insights into the long-term relevance of routine ED-based mental health screenings, which will inform decisions on the role of EDs in mental health care.

## METHODS

### Study Design, Setting, and Population

This longitudinal prospective observational study enrolled a convenience sample of adult patients (≥ 18 years of age) presenting with somatic (non-psychiatric) chief complaints to the EDs of two urban academic medical centers between July 2023–September 2023. During this period, the two EDs recorded a combined total of 37,898 adult visits, and 188 patients were enrolled. Institutional review board approval was obtained at both study sites (University of Chicago Biological Sciences Division Institutional Review Board [BSD IRB] Protocol IRB23-0849; Indiana University IRB Protocol IRB19235). Eligibility was limited to patients anticipated to be discharged from the ED, as determined by the treating clinician during routine clinical care at the time of screening.

### Study Protocol

To mitigate bias we used a convenience sampling strategy with a quasi-random sampling approach to identify ED patients for screening. Sampling sessions were conducted during limited daytime hours based on research staff availability; overnight enrollment was not performed. At the start of each sampling session, a research coordinator (RC) printed the ED patient census from the electronic health record (EHR) system. A random number digit (0–9) was generated (www.random.org) and matched to the last digit of a patient’s age to create a pre-screening list. Patients on the pre-screening list were then approached for screening in the order of their triage time. The process was repeated once all patients in the ED on the pre-screening list were approached.

The RC then verified patient stability and anticipated discharge from the ED with the treating clinician. Eligibility was limited to adults with non-psychiatric presentations who were stable, anticipated for discharge, and able to complete electronic surveys. Detailed inclusion and exclusion criteria are presented in Table 1. Eligible patients provided electronic written informed consent via Research Electronic Data Capture (REDCap, hosted at University of Chicago and Indiana University School of Medicine) and then completed the Computerized Adaptive Test for Suicide Severity (CAT-SS) suicide-risk screening tool.^18^ A “moderate” or “severe” score prompted immediate notification of the clinical team and termination of screening. Patients with a “low,” score proceeded to health literacy assessment (Rapid Estimate of Adult Literacy in Medicine, Revised **[**REALM-R]).^19^ Those meeting all criteria were enrolled in the study.

### Measurements

During the index visit, the RC collected sociodemographic and medical history information, and the participant completed baseline depression and anxiety assessments, as detailed below. Except in cases where suicidality was disclosed or detected during screening, treating clinicians were blinded to the outcomes of all survey assessments. The principal investigator and designated research staff were not blinded to the results. The RC collected demographic information and past medical history directly from participants during the index ED visit using REDCap surveys. See Table 2 for description of all screening tests used.

For depression and anxiety screening, patients first completed the Patient Health Questionnaire (PHQ)-8 and Generalized Anxiety Disorder-7 (GAD-7) via REDCap on a study tablet.^20^,^21^ They then proceeded to the CAT-MH suite (Adaptive Testing Technologies, Inc, Chicago, IL), limited in this study to the Computerized Adaptive Diagnostic screen for Major Depressive Disorder (CAD-MDD), the Computerized Adaptive Test-Depression Inventory (CAT-DI), and the Computerized Adaptive Test for Anxiety (CAT-ANX) assessments.^22^,^23^ Accessed via a secure, Health Insurance Portability and Accountability Act (HIPAA)-compliant platform, the CAT-MH system did not receive any protected health information. Instead, REDCap generated a study-specific participant identifier (eg, MCAT500, MCAT501) to maintain confidentiality. Patients could complete the assessments in either text-only or text-plus-audio format.

After completing the initial surveys, the RC confirmed the participant’s preferred contact details and emergency contact. The participant then received an email with a link to a $10 electronic gift card and was reminded that follow-up surveys would be sent at 1-, 2-, and 4-weeks post-ED discharge to complete the PHQ-8, GAD-7, and CAT-MH. At each follow-up point, patients received an email with a secure link to REDCap, where they logged in using the last four digits of their phone number. Upon completing the surveys, they received a confirmatory email/message with a link to a $10 electronic gift card (at the completion of each time point). Patients had up to six days to complete each set of surveys, with up to two email/message reminders and, if needed, one phone call reminder. While survey results were not shared, patients received a standardized resource list for managing anxiety and depression upon completing the surveys. Based on the structure and length of the demographic items, literacy screening, PHQ-8, GAD-7, and the adaptive CAT-MH assessments, a complete baseline session typically requires about 15–20 minutes to complete.

### Statistical Analysis

Descriptive statistics summarized baseline participant characteristics, with categorical variables reported as frequencies and percentages and continuous variables as means and standard deviations. The median time and interquartile range (IQR) for completing the Computerized Adaptive Tests (CAT-MH, PHQ-8, GAD-7) were also reported to assess testing efficiency in the ED.

A linear mixed-effects regression model assessed the stability of depression and anxiety severity measures from baseline to 30-day post-discharge, accounting for repeated measurements by including a random intercept for each participant. A linear trend was included to evaluate changes over time, and we used the intraclass correlation coefficient (ICC), calculated by dividing the intercept variance by the total variance, to assess test-retest reliability for continuous scores. Based on a 95% CI, ICC values were classified as poor (< 0.5), moderate (0.5–0.75), good (0.75–0.90), or excellent (> 0.90).^24^–^26^ The Cohen kappa coefficient assessed agreement (consistency) for the binary CAD-MDD outcome across the measurement occasions.^24^

Our mixed-effects model handled missing data and irregularly spaced measurement occasions, using all available data from each subject under the missing at random assumption in which assuming missingness is assumed to be ignorable conditional on covariates in the model and the measured outcomes for each individual (see Hedeker and Gibbons, 2006, *Longitudinal Data Analysis*, Wiley, Hoboken NJ). This allowed for valid inferences without imputing missing values.^27^ A chi-square test compared the longitudinal completion rates between MDD-positive vs MDD-negative groups and the normal/mild anxiety vs moderate/severe anxiety groups. We conducted all analyses using Python 3.1.3.0, released October 7, 2024 (Python Software Foundation, Wilmington, DE),^28^

## RESULTS

**Figure 1:**
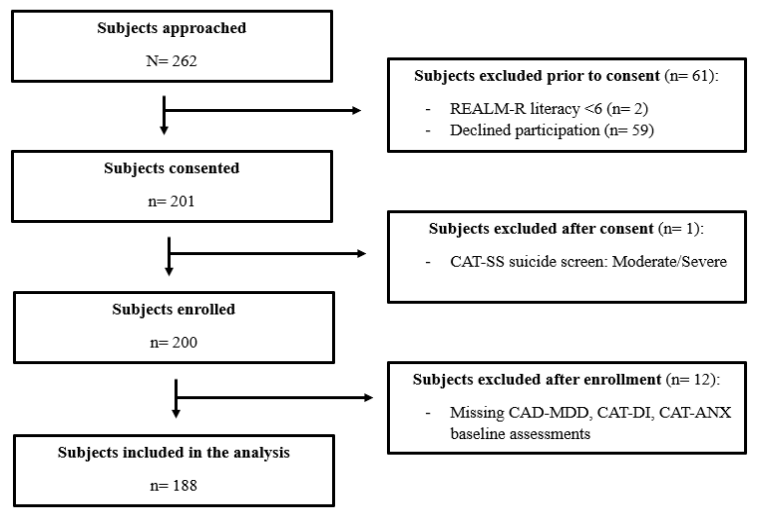
CONSORT flow diagram of enrollment and follow-up in a study of depression and anxiety trajectories among emergency department patients with somatic complaints. *CAT-SS*, Computerized Adaptive Test for Suicide Severity; *CAD-MDD*, Computerized Adaptive Diagnostic Test for Major Depressive Disorder; *CAT-DI*, Computerized Adaptive Test-Depression Inventory; CAT-ANX, Computerized Adaptive Test for Anxiety; CONSORT, Consolidated Standards of Reporting Trials; *REALM-R*, Rapid Estimate of Adult Literacy in Medicine, Revised.

During the study period, RCs approached 262 patients for enrollment (Figure 1). Of these, 59 declined to participate, two did not meet literacy criteria, and one screened moderate/severe for suicide risk, leaving 200 enrolled participants. An additional 12 patients were excluded for missing CAD-MDD, CAT-DI, and CAT-ANX baseline assessments, leaving 188 patients in the analytic sample, representing 72% of those approached.

Response rates to the Computerized Adaptive Tests decreased over time, with 42 responses at week 1, 41 at week 2, and 27 at week 4. Median completion times were 29 seconds (IQR 20) for CAD-MDD, 46.5 seconds (35.5) for CAT-DI, and 61 seconds (46.5) for CAT-ANX. These short times reflect the adaptive nature of the CAT-MH instruments, which administer only selected items rather than the full item banks. Results were as follows: 23.4% of patients screened positive for MDD (CAD-MDD); 9% for moderate/severe depression (CAT-DI); and 18.1% for moderate/severe anxiety (CAT-ANX). Depression and anxiety symptoms were positively correlated (r = 0.895, *P* < .001). Demographic characteristics are reported in Table 3.

Table 4 displays the mean and standard deviation for CAD-DI, CAT-ANX, PHQ-8, and GAD-7. Across 30 days, mean depression and anxiety scores showed no significant linear trends, indicating that mean scores did not change systematically over time (*P* values = .11–.66). The ICCs for all measures were high, suggesting that most of the variance in scores was attributable to differences between individuals, with ICCs of 0.8447 (CAT-DI), 0.8341 (CAT-ANX), 0.7629 (PHQ-8), and 0.7652 (GAD-7). For depression as measured by CAD-MDD, Cohen kappa values compared to baseline were 0.74 at Week 1 (95% CI, 0.45–0.95), 0.62 at Week 2 (0.33–0.85), and 0.46 at Week 4 (0.07–0.78).

Participants with baseline major depressive disorder were more likely to complete follow-up assessments at weeks 2 and 4 (*P* = .04), whereas completion rates did not differ significantly by baseline anxiety status (Table 5).

## DISCUSSION

In this convenience sample of ED patients presenting with non-psychiatric complaints, we identified a high baseline prevalence of depression (23%) and anxiety (18%). These rates are consistent with prior ED studies, and significantly higher than those typically reported in adult primary care populations in the U.S.,^12^,^13^,^29^ reinforcing the ED as a critical point of contact for patients with unmet psychiatric needs. These high rates are concerning given their association with poor health outcomes, repeat visits, suicide risk, and long-term disability.^6^,^10^,^11^,^30^,^31^ Depression and anxiety were also strongly correlated, consistent with prior work underscoring importance of addressing both conditions together during screening.^32^ Integrating effective screening and referral in the ED remains challenging given the time-sensitive nature of emergency care, limited resources, and inadequate follow-up services.^11^,^33^ Nonetheless, the success of screening programs in other settings and the proven efficacy of treatments support the development of ED-based approaches.^10^,^11^,^33^

We acknowledge that ED care is inherently time- and resource-limited, and that additional screening must be weighed against competing clinical priorities. However, depression and anxiety are highly prevalent among ED patients and can be identified using brief, self-administered tools that require minimal clinician time. When integrated into existing workflows, ED-based mental health screening may represent a high-yield opportunity to identify untreated conditions and facilitate linkage to care without meaningfully detracting from emergent care.

Our longitudinal analyses demonstrated strong within-subject reliability for both depression and anxiety severity scores over 30 days, with ICC values above accepted thresholds for reliability. 21,24,25 The Cohen kappa showed substantial agreement for depression at Week 1 (k = 0.738) and moderate-to-good agreement at Weeks 2 (k = 0.616) and 4 (k = 0.455). Although kappa declined over time, likely reflecting smaller samples and normal response variability, the overall pattern suggests persistent depressive symptoms, which merit clinical attention even without a CAD-MDD diagnosis.^34^ These findings suggest that ED-based screening may identify persistent mental health conditions and may offer an opportunity for early recognition and linkage to care.

In addition, the Computerized Adaptive Tests (CAT-DI and CAT-ANX) outperformed traditional measures such as the PHQ-8 and GAD-7, providing more precise severity estimates with fewer items.^35^,^36^ Median completion times were under one minute, and the self-administered format can improve disclosure of sensitive symptoms,^37^,^38^ making them particularly well suited for the time-pressured ED setting. Although not diagnostic, these tools are clinically actionable, as elevated scores are frequently used to guide treatment initiation in primary care settings.^39^ Notably, the CAD-MDD was developed as a diagnostic tool rather than a screening test, and it has shown strong agreement with the SCID,^23^,^35^ supporting its use as a practical diagnostic proxy when full psychiatric interviews are not feasible in the ED.

Follow-up response rates in our study were low (22% at week 1, 22% at week 2, and 14% at week 4), which constrains the strength of conclusions regarding symptom trajectories and introduces the potential for nonresponse selection bias.^40^,^41^ Notably, patients who screened positive for depression were more likely to complete follow-up assessments at Weeks 2 and 4, suggesting that individuals with greater psychiatric burden may remain more engaged. This pattern is consistent with prior reports of higher retention among symptomatic patients.^33^ Low overall response rates may have been compounded by our reliance on email-based REDCap links, as previous studies show lower responsiveness to email compared with text- or phone-based approaches, particularly among socioeconomically disadvantaged groups.^42^ Future work should explore alternative strategies such as HIPAA-compliant text messaging or secure patient portal communication, both of which have demonstrated higher adoption and patient engagement,^43^,^43^–^45^ and may be most effective when implemented together as part of a multimodal follow-up strategy. 40,41

Taken together, our findings highlight the ED as a critical setting for early psychiatric recognition. Screening alone is insufficient; benefit will depend on pairing identification with referral and timely intervention. Stepped-care frameworks may help tailor treatment intensity to symptom severity, while digital solutions such as telepsychiatry and mobile health application could extend access and mitigate workforce shortages.^46^,^47^ Our results also suggest that at home, self-administered follow-up assessments are feasible and may provide ongoing opportunities for monitoring beyond the ED encounter. Future studies should test whether systemic ED-based screening, coupled with treatment initiation or referral, improves long-term outcomes. Larger and more diverse populations will be needed to establish the effectiveness, scalability and sustainability of ED-initiated mental health interventions.

## LIMITATIONS

While these findings provide insight into the mental health burden in the ED, several factors limit their interpretation. The use of a convenience sample reduces representativeness, as patients who participated may differ from those who declined or were excluded, and non-responders were not characterized in detail. To mitigate this limitation, patient selection within each session was randomized, reducing the risk of systematic bias.

Restricting eligibility to patients anticipated for discharge and excluding non-English-speaking patients may have limited generalizability by excluding individuals with more severe conditions and potentially different baseline symptom levels or psychiatric trajectories. Screenings were also conducted during limited hours, excluding overnight patients; however, this group accounts for only about 10% of the ED population. Recruitment from two urban, academic centers further limits generalizability to other ED settings.

In addition, follow-up response rates were low, which limits the strength of conclusions regarding symptom trajectories; reliance on phone number-based identification may have further introduced selection bias if patients without consistent phone access were less likely to participate. The four-week follow-up period may not capture longer term changes in depression and anxiety beyond the acute post-discharge period, and recall of prior responses during follow-up assessments may also have biased results toward showing little or no change over time. Neither were we able to account for repeat ED visits during the follow-up interval, which could have influenced symptom trajectories if patients experienced new or recurrent acute illness.

The study also lacked detailed information on prior psychiatric treatment and established risk factors for depression and anxiety, such as frequent ED visits, smoking, or comorbidities including asthma and arthritis.^9^ Prior work has shown that depression in ED patients is more common among middle-aged women, individuals with lower socioeconomic or educational backgrounds, and those reporting anxiety or chronic fatigue.^29^ Incorporating such variables could help refine screening strategies and improve the identification of patients at highest risk.

## CONCLUSION

This study highlights the substantial burden of depression and anxiety among ED patients presenting with non-psychiatric complaints. Among participants who completed follow-up assessments, severity score remained stable over 30 days, suggesting that these symptoms may reflect ongoing conditions rather than temporary responses to acute illness. Given the limited number of follow-up assessments, these findings should be interpreted cautiously. Future studies should focus on improving follow-up response and testing whether ED-based identification and treatment of depression and anxiety can improve patient outcomes.

## Figures and Tables

**Table 1:** Inclusion and exclusion criteria in a study of depression and anxiety trajectories among emergency department patients with somatic complaints.

Inclusion Criteria Adult ED patients (≥ 18 years of age) Emergency Severity Index triage score from 3–5 (5 indicates non-urgent) Somatic, non-mental health chief complaint Able to demonstrate English reading literacy of at least 8th grade level (REALM-R ≥ 6) Willing to participate and able to give written informed consent Owns a smartphone with data plan or home computer Anticipated discharge from the ED as predicted by physician Exclusion Criteria Prior enrollment in the study Documented dysthymia or Axis II diagnoses Under involuntary detention for psychiatric assessment Prisoners Lack of decisional capacity to participate in informed consent as reported by the treating physician (e.g., active psychosis, hallucinations, intoxication, dementia, delirium, developmental delay) Active suicidality as indicated by CAT-SS suicide risk of “moderate” or “severe,” as determined by the clinician or disclosed during screening Clinical instability as judged by clinician Non-English speaking

*CAT-SS*, Computerized Adaptive Test for Suicide Severity; *ED*, emergency department; *REALM-R*, Rapid Estimate of Adult Literacy in Medicine, Revised.

**Table 2:** Screening tools for depression, anxiety, literacy, and suicide risk used in a study of emergency department patients with somatic complaints.

Test Name	Description
Computerized Adaptive Test-Suicide Scale (CAT-SS)	The CAT-SS is an adaptive measure, comprised of 111 items, that dimensionally measures suicide risk severity on a 100-point scale with 5 points of precision.^18^ The scores are also categorized to yield categories of low, moderate, and high risk. This survey is administered via the CAT-MH system. Because the CAT-SS is based on item response theory, the algorithm selects only the most informative questions for each participant, so individuals typically complete about 10 items rather than the full 111.
Rapid Estimate of Adult Literacy in Medicine, Revised (REALM-R)	The REALM-R is a word recognition test of 11 items used to identify people at risk for poor health literacy.^19^ This survey is administered by the research staff.
Computerized Adaptive Diagnostic Test for Major Depressive Disorder (CAD-MDD)	The CAD-MDD is a proprietary computerized adaptive depression screening tool that adapts to patient responses to questions about depression by asking the most diagnostically informative question out of an item bank of almost 400 items.^23^ A prior study showed that the CAD-MDD was on average shorter than the PHQ-9 (an average of 4 vs 9 items), and that overall sensitivity and specificity for the CAD-MDD was 0.95 and 0.87, respectively, compared to 0.70 and 0.91 for the PHQ-9, as compared to the Structural Clinical Interview for DSM-IV (SCID).^23^ This survey is administered directly via the CAT-MH system.
Computerized Adaptive Test-Depression Inventory (CAT-DI)	The CAT-DI is a proprietary computerized adaptive dimensional severity measure for depression.^17^ The CAT-DI uses a bank of 389 depression items. The item bank was originally calibrated using a multidimensional item-response theory model. An adaptive test was then constructed that uses the item parameters to select an optimal small subset of items from the item bank that is tailored to a specific participant’s depression severity, which is dynamically estimated as the participant responds to successive test items. The process continues until the uncertainty in this severity score estimate drops below a pre-specified level (eg, 5 points on a 100-point scale). A prior study has shown that an average of 12 items and a median time of 137 seconds had a correlation of r = 0.95 with the 389 total item bank score.^22^ The resulting severity score has outstanding predictive accuracy for a DSM-IV (SCID) diagnostic categories of none, minor depression (including dysthymia), and MDD. The CAT-DI categorized responses as normal (< 50), mild symptoms (50–65), moderate symptoms (66–75), and severe symptoms (> 75).^24^ This survey is administered via the CAT-MH system.
Computerized Adaptive Test for Anxiety (CAT-ANX)	The CAT-ANX is a proprietary computerized adaptive dimensional severity measure for anxiety. It accurately predicts generalized anxiety disorder severity scores using an average of 12 items per participant in less than 3 minutes from a bank of 431 items.^23^ The CAT-ANX scores range from 0–100 and are grouped as normal (< 35), mild (35–50), moderate (51–65), and severe symptoms (> 65).^23^ This survey is administered via the CAT-MH system.
Patient Health Questionnaire 8 (PHQ-8)	The PHQ-8 is an 8-question, self-reported screening tool for depression screening and symptom severity.^20^ It consists of eight questions that ask about common symptoms of depression. Each question is scored on a scale from 0–3, with a total score ranging from 0–27. Higher scores indicate more severe depression symptoms. This survey was administered via REDCap.
Generalized Anxiety Disorder-7 (GAD-7)	The GAD-7 survey tool is a widely used and validated screening tool used to measure the severity of generalized anxiety disorder (symptoms in adults.^21^ It consists of seven questions that ask about common anxiety. Each question is scored on a scale from 0–3, with a total score ranging from 0–21. Higher scores indicate more severe anxiety symptoms. This survey was administered via REDCap.

*CAT-MH*, Computerized Adaptive Testing for Mental Health; *DSM-IV*, *Diagnostic and Statistical Manual of Mental Disorders*, 4^th^ Ed.; *SCID*, Structured Clinical Interview for DSM-IV; *MDD*, major depressive disorder; *REDCap*, Research Electronic Data Capture.

**Table 3:** Baseline sociodemographic and clinical characteristics of participants in a study of depression and anxiety trajectories among emergency department patients with somatic complaints (N = 188).

		Frequency	%
Age, mean (SD)	42.8 (16.5)		
Sex	Female	126	67%
	Male	60	32%
	Other	2	1%
Ethnicity	Hispanic/Latino	15	8%
	Non-Hispanic/Latino	170	90%
Race	Black	111	59%
	White	65	35%
	Other	12	6%
Marital Status	Single	123	65%
	Married	42	22%
	Separated	3	2%
	Divorced	14	7%
	Widowed	6	3%
Education	High school/GED	87	46%
	Some college	54	29%
	Graduated college	31	16%
	Graduate/professional school	16	9%
Employment	Full-time	86	46%
	Part-time	18	10%
	Student and working	6	3%
	Student and not working	6	3%
	Homemaker	3	2%
	Unemployed	36	19%
	Retired	15	8%
	Disabled	18	10%
Healthcare Data	PCP^a^	140	74%
Comorbid diagnosis^b^	Asthma, emphysema, or chronic bronchitis	45	24%
	Hypertension	63	34%
	Diabetes	33	18%
	Arthritis or rheumatism	47	25%
	Heart attack, angina, heart failure or other heart disease	24	13%
	Stroke, seizures, Parkinson disease, or other neurological condition	20	11%
	Liver disease	5	3%
	Kidney disease	16	9%
	Cancer diagnosed or treated in the prior 3 years	11	6%
	Anxiety	66	35%
	Depression	60	32%
	Post-traumatic stress disorder	34	18%

a Percentage of participants who have a PCP (primary care physician).

b Refers to a chronic health problem diagnosed by a healthcare worker in the prior 3 years.

*SD*, standard deviation; *GED*, General Educational Development.

**Table 4:** Mean and standard deviation of depression and anxiety severity scores over 30 days in a study of emergency department patients with somatic complaints.

Time-point		Baseline	Week 1	Week 2	Week 4
Test	CAT-DI^a^	32.25 (21.65)	30.24 (22.58)	38.17 (29.79)	38.96 (29.22)
	CAT-ANX^b^	26.71 (24.07)	25.32 (25.37)	20.93 (32.92)	35.34 (33.19)
	PHQ-8	6.55 (5.56)	6.71 (6.79)	6.87 (6.64)	7.24 (6.91)
	GAD-7	5.46 (5.11)	5.77 (5.64)	6.06 (5.98)	6.40 (6.27)

a CAT-DI scores range from 0–100 and are grouped as normal (< 50), mild symptoms (50–65), moderate symptoms (66–75), and severe symptoms (> 75).

b CAT-ANX scores range from 0–100 and are grouped as normal (< 35), mild (35–50), moderate (51–65), and severe symptoms (> 65).

*CAT-DI*, Computerized Adaptive Test-Depression Inventory; *CAT-ANX*, Computerized Adaptive Test for Anxiety; *PHQ-8*, Patient Health Questionnaire-8; *GAD-7*, Generalized Anxiety Disorder-7.

**Table 5:** Completion rates of longitudinal follow-up assessments by baseline depression and anxiety status in a study of emergency department patients with somatic complaints.

	Week 1	Week 2	Week 4
MDD negative completion %	21.53%	18.06%	11.11%
MDD positive completion %	25.00%	34.09%	25.00%
Chi-square	0.077	4.185	4.217
*P* value	.78	.04	.04
Normal/mild anxiety completion %	20.26%	18.95%	11.76%
Moderate/severe anxiety completion %	23.53%	35.29%	26.47%
Chi-square	0.036	3.437	3.752
*P* value	.85	.06	.05

*MDD*, major depressive disorder.
